# Inhibition of Inflammasome-Dependent Interleukin 1β Production by Streptococcal NAD^+^-Glycohydrolase: Evidence for Extracellular Activity

**DOI:** 10.1128/mBio.00756-17

**Published:** 2017-07-18

**Authors:** Dóra Hancz, Elsa Westerlund, Benedicte Bastiat-Sempe, Onkar Sharma, Christine Valfridsson, Lena Meyer, John F. Love, Maghnus O’Seaghdha, Michael R. Wessels, Jenny J. Persson

**Affiliations:** a Immunology Section, Department of Experimental Medical Sciences, Lund University, Lund, Sweden; b Division of Infectious Diseases, Boston Children’s Hospital, and Department of Pediatrics, Harvard Medical School, Boston, Massachusetts, USA; New York University School of Medicine

**Keywords:** *Streptococcus pyogenes*, host-pathogen interactions, immune regulation, innate immunity

## Abstract

Group A *Streptococcus* (GAS) is a common human pathogen and the etiologic agent of a large number of diseases ranging from mild, self-limiting infections to invasive life-threatening conditions. Two prominent virulence factors of this bacterium are the genetically and functionally linked pore-forming toxin streptolysin O (SLO) and its cotoxin NAD^+^-glycohydrolase (NADase). Overexpression of these toxins has been linked to increased bacterial virulence and is correlated with invasive GAS disease. NADase can be translocated into host cells by a SLO-dependent mechanism, and cytosolic NADase has been assigned multiple properties such as protection of intracellularly located GAS bacteria and induction of host cell death through energy depletion. Here, we used a set of isogenic GAS mutants and a macrophage infection model and report that streptococcal NADase inhibits the innate immune response by decreasing inflammasome-dependent interleukin 1β (IL-1β) release from infected macrophages. Regulation of IL-1β was independent of phagocytosis and ensued also under conditions not allowing SLO-dependent translocation of NADase into the host cell cytosol. Thus, our data indicate that NADase not only acts intracellularly but also has an immune regulatory function in the extracellular niche.

## IMPORTANCE

In the mid-1980s, the incidence and severity of invasive infections caused by serotype M1 GAS suddenly increased. The results of genomic analyses suggested that this increase was due to the spread of clonal bacterial strains and identified a recombination event leading to enhanced production of the SLO and NADase toxins in these strains. However, despite its apparent importance in GAS pathogenesis, the function of NADase remains poorly understood. In this study, we demonstrate that NADase inhibits inflammasome-dependent IL-1β release from infected macrophages. While previously described functions of NADase pertain to its role upon SLO-mediated translocation into the host cell cytosol, our data suggest that the immune regulatory function of NADase is exerted by nontranslocated enzyme, identifying a previously unrecognized extracellular niche for NADase functionality. This immune regulatory property of extracellular NADase adds another possible explanation to how increased secretion of NADase correlates with bacterial virulence.

## INTRODUCTION

Group A *Streptococcus* (GAS) (*Streptococcus pyogenes*) is a major human pathogen responsible for a wide array of diseases with different levels of severity, ranging from self-limiting mucosal and skin infections to life-threatening septic and/or necrotic conditions requiring high doses of intravenous antibiotics and at times surgical removal of infected tissues (1). It has been estimated that GAS causes at least 700 million superficial infections and 650,000 invasive infections worldwide each year (2).

GAS is a Gram-positive bacterium with a chiefly extracellular lifestyle, and its ability to infect virtually any human tissue can likely be ascribed to the expression of an impressive set of virulence factors (1, 3). Among these virulence factors are two genetically and functionally linked secreted toxins, the pore-forming cytolysin streptolysin O (SLO) and the β-NAD^+^ glycohydrolase or NADase (also known as SPN for *Streptococcus pyogenes* NADase) which hydrolyzes β-NAD^+^ (here referred to as NAD^+^) into nicotinamide (NAM) and ADP-ribose (ADPR) (4). Notably, gaining a genomic region mediating increased expression of SLO and NADase correlates with an increase in invasive GAS disease (5–8) emphasizing the role for these toxins in GAS pathogenesis.

NAD^+^ is an essential coenzyme in many metabolic and energy-producing reactions and a substrate in several enzymatic processes. Enzymes using NAD^+^ as a substrate are common in both eukaryotic and prokaryotic cells, and many of these enzymes hydrolyze NAD^+^ to generate NAM and ADPR. Depending on the enzyme involved, the ADPR moiety can be further transformed into cyclic ADPR (cADPR) or transferred to a target protein (ADP-ribosylation) (9). In addition, the free enzymatic products, NAM and ADPR or cADPR, are compounds with known effects, including inhibition of proinflammatory cytokine production from monocytes (10) and stimulation of cellular Ca^2+^ flux (11). Recent data suggest that streptococcal NADase is a strict hydrolase, thus generating free NAM and ADPR only (12). Streptococcal NADase was long the only known bacterial toxin of its kind, but *Mycobacterium tuberculosis* was recently demonstrated to produce a NAD^+^-hydrolase with genetic homologs present in many additional bacterial species, indicating that such toxins may be of general importance in microbial pathogenesis (13).

SLO belongs to a family of cholesterol-dependent cytolysins, capable of forming large pores in host cell membranes (14). When GAS bacteria are adherent to a host cell, SLO is also able to specifically deliver NADase across the host cell membrane through a pore-independent process known as cytolysin-mediated translocation (CMT) (15, 16). SLO and NADase play significant and functionally linked roles in GAS pathogenesis through their ability to protect intracellularly located GAS from degradation by autophagy and their effect on phagolysosomal acidification, intracellular pools of NAD^+^, and host cell death (17–24). For the work reported here, it is of particular interest that SLO activates the innate immune response of the host (25).

Innate immune mechanisms constitute our first line of defense against invading microbes, and the nature of triggered responses may profoundly impact microbial survival and ability to spread. Once a microbe has penetrated the physical barriers of the host, recognition is typically performed by pattern recognition receptors (PRRs), such as Toll-like receptors (TLRs) or nucleotide binding domain and leucine rich repeat-containing proteins (NLRs). This recognition may result in multiple responses pertaining to the production and release of proinflammatory cytokines. One such cytokine is the multifaceted interleukin 1β (IL-1β), which exerts both local and systemic effects. Not much is known about the precise role for IL-1β in GAS infections, but recent data indicate that patients treated with the IL-1 receptor (IL-1R) antagonist anakinra have significantly increased risk of developing necrotizing fasciitis, suggesting that IL-1 signaling has a protective role in this destructive tissue disease (26). IL-1β is produced as an inactive proform, pro-IL-1β, which is subsequently cleaved to generate mature IL-1β, a cleavage that can be performed by a number of proteases (27). In particular, IL-1β maturation can be performed by the cysteine protease caspase-1 within the cytosolic complexes known as inflammasomes. In addition to caspase-1, inflammasomes typically include a “sensor protein,” such as Nlrp3, and the bimodular adaptor protein ASC (apoptosis-associated speck-like protein containing a CARD [caspase activation and recruitment domain]) (28). Interestingly, the Nlrp3 inflammasome can be activated by bacterial cytolytic toxins (29), including streptococcal SLO (25). Nlrp3 inflammasome activation can be triggered by a number of stimuli, and although this inflammasome is the most extensively studied, the exact mechanism by which SLO, or any other stimulus, activates Nlrp3 is unclear. It has been convincingly shown that activation of the Nlrp3 inflammasome by pore-forming toxins depends on K^+^ efflux (30); however, the mechanism by which ion flux links to Nlrp3 activation remain elusive.

In this study, we used a set of isogenic GAS mutants and a macrophage infection model and report that streptococcal NADase inhibits the innate immune response by decreasing inflammasome-dependent IL-1β release. Remarkably, our data indicate that this effect is triggered by NADase that is not translocated, implying that this toxin not only acts intracellularly but also exerts functions in an extracellular niche.

## RESULTS

### Streptococcal NADase reduces the level of IL-1β secreted from macrophages.

**FIG 1:**
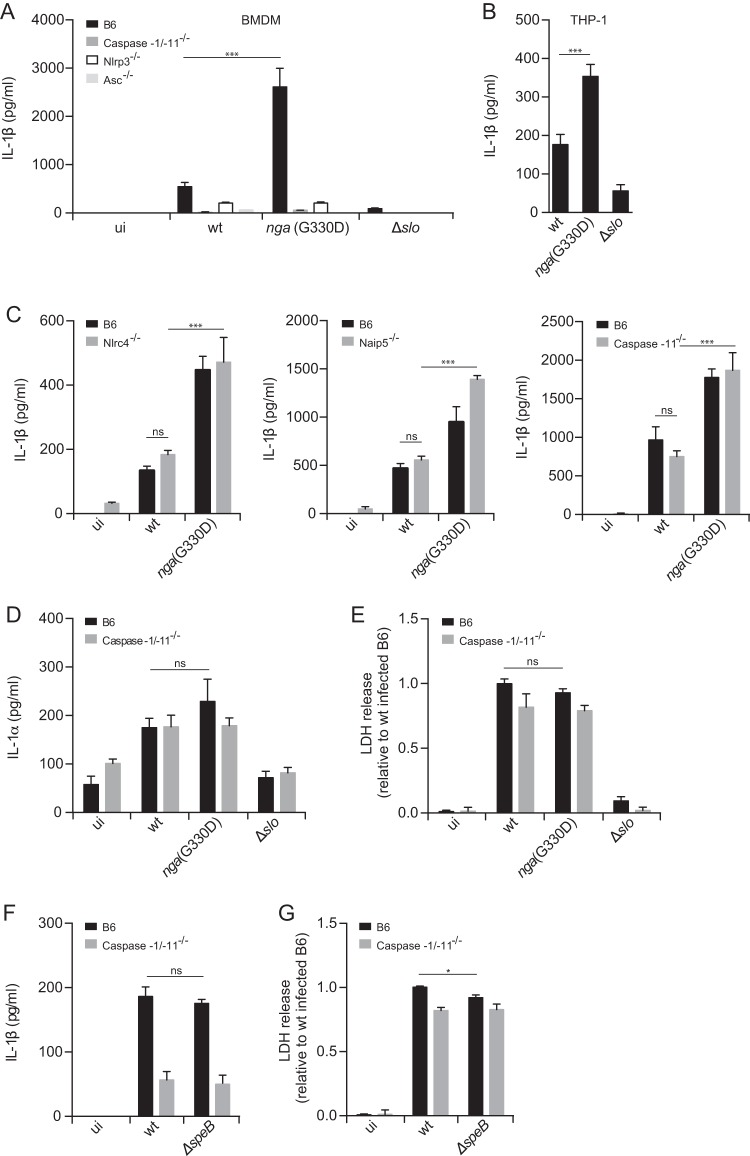
Streptococcal NADase regulates inflammasome-dependent release of IL-1β. (A) BMDMs of the indicated genotypes were primed with LPS and infected with wt, *nga*(G330D), or Δ*slo* streptococci. ui, uninfected. (B) Differentiated and LPS-primed THP-1 cells were infected with wt, *nga*(G330D), or Δ*slo* bacteria. (C to G) LPS-primed BMDMs were infected with wt, *nga*(G330D), Δ*slo*, or Δ*speB* GAS. Supernatants were analyzed, and secretion of IL-1β (A, B, C, and F) or IL-1α (D) was determined by ELISA or cytometric bead array (CBA), respectively. (E and G) Cell death was evaluated by measuring LDH release. Values were normalized to LDH release induced by wt bacterial infection of B6 macrophages. Graphs show means plus standard deviations (SD) (error bars) for triplicate samples and are representative of at least three independent experiments. Values that are significantly different are indicated by asterisks as follows: ***, *P* ≤ 0.001; *, *P* ≤ 0.05. Values that are not significantly different (ns) are indicated.

It has been shown previously that GAS activates the Nlrp3 inflammasome in murine bone marrow-derived macrophages (BMDMs) in a SLO-dependent manner (25). Because SLO can promote the translocation of NADase through a cytolysin-mediated translocation (CMT) (15), we were interested in analyzing whether NADase was involved in inflammasome activation by GAS. Analysis of this question with mutants completely lacking NADase raised a problem, because NADase and SLO are known to physically interact, and lack of NADase may have direct effects on SLO stability (19, 31; M. R. Wessels, unpublished data). To avoid this problem, we took advantage of a mutant strain harboring a point mutation (specifically, a G-to-D change at position 330) in the *nga* gene [*nga*(G330D)], rendering NADase enzymatically inactive but fully functional for CMT (21, 32), and expressing SLO at levels similar to those of the wild-type (wt) bacteria (see Fig. S1 in the supplemental material). Unexpectedly, we found that lipopolysaccharide (LPS)-primed BMDMs (Fig. 1A and Fig. S2A), as well as similarly primed macrophages derived from the human monocytic cell line THP-1 (Fig. 1B), infected with the *nga*(G330D) mutant strain secrete significantly increased levels of IL-1β than cells infected with the wt bacteria (wt-infected cells) do, suggesting a role for this toxin as a negative regulator of SLO-mediated IL-1β release. Western blot analysis of supernatants from infected cells confirmed an increase of released mature IL-1β from *nga*(G330D) mutant-infected cells compared to wt-infected cells (Fig. S3A), and cells infected with wt or *nga*(G330D) bacteria released low and comparable levels of pro-IL-1β (Fig. S3B). We thus conclude that the measured increase of IL-1β induced by the *nga*(G330D) strain is indeed due to release of mature cytokine rather than the unprocessed proform. Importantly, active NADase toxin does not seem to have any direct effects on the IL-1β protein (Fig. S4). In line with our observations on IL-1β, BMDMs infected with the *nga*(G330D) mutant released increased levels of IL-18 compared to wt-infected cells (Fig. S2B), which suggests that similar regulating mechanisms act on both cytokines. In agreement with previously published data, a strain deficient for SLO expression (Δ*slo*) did not activate the inflammasome, and IL-1β release induced by SLO competent bacteria in BMDMs was dependent on the inflammasome components caspase-1, Nlrp3, and ASC (Fig. 1A), but not the sensors Naip5 (NLR family, apoptosis inhibitory protein 5) or Nlrc4 (NLR family, CARD domain containing 4) (Fig. 1C) (25). Moreover, the increased IL-1β secretion mediated by the *nga*(G330D) strain was dependent on the same inflammasome components as IL-1β secretion mediated by wt bacteria, showing that these two bacterial strains similarly activate a Nlrp3 inflammasome (Fig. 1A and C).

10.1128/mBio.00756-17.1 FIG S1  Expression levels of SLO and NADase from wt and mutant GAS strains. Overnight cultures of GAS were reinoculated in fresh THY and grown until they reached an OD_600_ of 1.15. Supernatants were harvested, and levels of secreted NADase and SLO were analyzed by Western blotting. Bacteria from the same culture were lysed, and M1 protein expression was assessed as the loading control. The figure shows results of one experiment representative of four independent experiments. Download FIG S1, EPS file, 5.0 MB. Copyright © 2017 Hancz et al. 2017 Hancz et al. This content is distributed under the terms of the Creative Commons Attribution 4.0 International license.

10.1128/mBio.00756-17.2 FIG S2  Cytokine and LDH release kinetics from infected macrophages. IL-1β (A), IL-18 (ELISA; Invitrogen) (B), or LDH (C) release was measured upon wt, *nga*(G330D), or Δ*slo* GAS infection of LPS-primed BMDMs at 1.5 and 4.5 h postinfection (hpi). FliC was transfected with Lipofectamine 2000 as a positive control for IL-18 induction. Graphs show means ± SD for triplicate samples and are representative of at least three independent experiments. Download FIG S2, EPS file, 2.2 MB. Copyright © 2017 Hancz et al. 2017 Hancz et al. This content is distributed under the terms of the Creative Commons Attribution 4.0 International license.

10.1128/mBio.00756-17.3 FIG S3  Macrophages infected with wt or *nga*(G330D) GAS mainly secrete mature IL-1β. (A) As IL-10^−/−^ BMDMs secrete increased levels of IL-1β upon inflammasome activation compared to B6 BMDMs (P. Gurung et al., Sci Rep **5**:14488, 2015, https://doi.org/10.1038/srep14488; also data not shown), we used IL-10^−/−^ cells to overcome detection threshold issues for Western blotting of IL-1β released from B6 BMDMs. LPS-primed IL-10^−/−^ BMDMs were infected with wt, *nga*(G330D), or Δ*slo* streptococci, and IL-1β was determined by ELISA or Western blotting (p17) in the supernatant. (B) Pro-IL-1β levels were evaluated by ELISA (Invitrogen) upon infection of LPS-primed BMDMs. Graphs show means ± SD for triplicate samples and is representative of three independent experiments. Download FIG S3, EPS file, 2.7 MB. Copyright © 2017 Hancz et al. 2017 Hancz et al. This content is distributed under the terms of the Creative Commons Attribution 4.0 International license.

10.1128/mBio.00756-17.4 FIG S4  Active NADase has no direct effects on the IL-1β protein. Recombinant IL-1β (rIL-1β) and cleared supernatant from *nga*(G330D) GAS-infected BMDMs were incubated in the presence of rNADase (3 to 300 nM), followed by IL-1β ELISA. The graph shows means ± SD for triplicate samples. Download FIG S4, EPS file, 1.8 MB. Copyright © 2017 Hancz et al. 2017 Hancz et al. This content is distributed under the terms of the Creative Commons Attribution 4.0 International license.

Because the caspase-1-deficient mouse strain used in this study is also deficient for caspase-11, it was important to exclude a role for caspase-11 in SLO-mediated activation of the inflammasome. Caspase-11-deficient and C57BL/6 (B6) macrophages secreted similar amounts of IL-1β in response to GAS infection, indicating that IL-1β secretion induced by SLO is completely caspase-1 dependent (Fig. 1C).

Synthesis of the proform of IL-1α is governed by similar transcriptional pathways as those of IL-1β, but its cleavage is independent of the inflammasome (33). Under certain conditions, however, IL-1α is dependent on the inflammasome for its release (33). This is not the case for GAS infection of BMDMs since caspase-1/-11-deficient and B6 cells secrete similar levels of IL-1α (Fig. 1D). Importantly, the wt and *nga*(G330D) strains induced comparable levels of IL-1α upon infection of macrophages (Fig. 1D), suggesting that increased IL-1β levels induced by the *nga*(G330D) strain is governed by a specific, likely posttranslational, mechanism.

Although NADase is a toxin with established effects on cell death (17, 19), we observed no differences in induced macrophage death after infection with wt or *nga*(G330D) bacteria (Fig. 1E and Fig. S2C), excluding the possibility that altered IL-1β levels are due to differences in cell viability after infection with the separate strains. Notably, the Δ*slo* strain did not induce cell death in infected BMDMs, indicating that in our system, SLO is the main toxin responsible for cytotoxicity (34). Caspase-1/-11^−/−^ BMDMs were only slightly less sensitive to cell death induced by GAS compared to B6 BMDMs (Fig. 1E), confirming previous studies suggesting that macrophage death induced by GAS is mainly nonpyroptotic in nature (34–36).

The streptococcal cysteine protease SpeB has previously been shown to directly cleave pro-IL-1β into its mature form (37), and a recent study implicates this virulence factor in inflammasome-independent IL-1β maturation in a murine model of GAS infection as well as in infection of murine BMDMs *in vitro* (26). Our findings implied that IL-1β release from GAS-infected macrophages *in vitro* is dependent on the inflammasome and streptococcal SLO. To further explore a possible involvement of other virulence factors, we employed an isogenic mutant strain lacking expression of the SpeB protein (Δ*speB*). This strain induced IL-1β levels from infected macrophages in a caspase-1-dependent manner similar to wt bacteria (Fig. 1F), suggesting that SpeB is not involved in caspase-1-independent processing of IL-1β during infection of BMDMs. In addition, we observed only a slight difference in cytotoxicity between the Δ*speB* and wt strains after infection of B6 BMDMs (Fig. 1G), and the ability of these strains to induce cell death was independent of caspase-1. Collectively, the findings described above indicate a previously unknown role for NADase as a negative regulator of inflammasome-dependent IL-1β release, identifying a novel immune evasion mechanism employed by GAS.

### Inflammasome activation and modulation of IL-1β levels are independent of phagocytosis.

**FIG 2:**
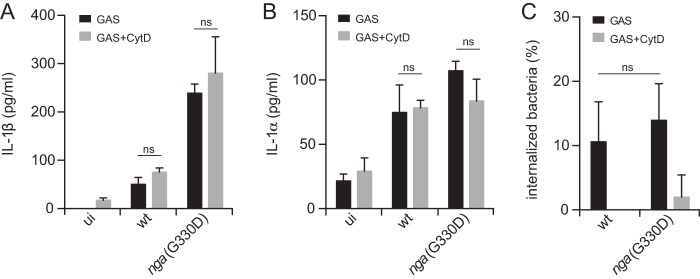
Inflammasome activation and regulation are independent of phagocytosis. LPS-primed BMDMs were infected with GAS in the presence of cytochalasin D (CytD). Supernatants were analyzed for the secretion of IL-1β (A) and IL-1α (B) by cytometric bead array (CBA). (C) The proportion of internalized bacteria was determined following gentamicin treatment. Graphs show means plus SD for triplicate samples and are representative of at least three independent experiments. ui, uninfected.

Although GAS is an extracellular pathogen, intracellular survival in macrophages for extended periods has been demonstrated and put forward as an explanation for the occurrence of recurring infections in certain patients even after treatment with antibiotics (38). In light of this, we were interested in analyzing whether the observed modulation of inflammasome-dependent IL-1β release (Fig. 1A) originated from extra- or intracellularly located bacteria. Interestingly, blunting phagocytosis during infection using the actin polymerization inhibitor cytochalasin D (CytD) had no effect on levels of IL-1β induced by either strain (Fig. 2A), nor did CytD affect the amount of secreted IL-1α (Fig. 2B). Importantly, at the CytD concentration used in GAS infections, phagocytosis of GAS (Fig. 2C), or zymozan (Fig. S5), by BMDMs was efficiently inhibited. These data suggest that, as for the production of inflammasome-independent IL-1α induced by GAS, inflammasome-dependent IL-1β release and its modulation by NADase occur in the absence of phagocytosis and are provoked by extracellularly located bacteria. In addition, these results suggest that inflammasome activation by GAS is independent of the ability to modulate the actin cytoskeleton, which is in contrast to several other Nlrp3-activating stimuli (39).

10.1128/mBio.00756-17.5 FIG S5  Cytochalasin D dose dependently inhibits phagocytosis. BMDMs were incubated with zymosan for 1.5 h in the presence of increasing concentration (0.31 to 5 µg/ml) of cytochalasin D. The proportion of phagocytosed zymosan was determined using CytoSelect 96-well phagocytosis assay (zymosan substrate). The graph shows means ± SD for triplicate samples and is representative of three independent experiments. Download FIG S5, EPS file, 1.8 MB. Copyright © 2017 Hancz et al. 2017 Hancz et al. This content is distributed under the terms of the Creative Commons Attribution 4.0 International license.

### Reduction of IL-1β secretion by NADase occurs in the absence of toxin translocation.

**FIG 3:**
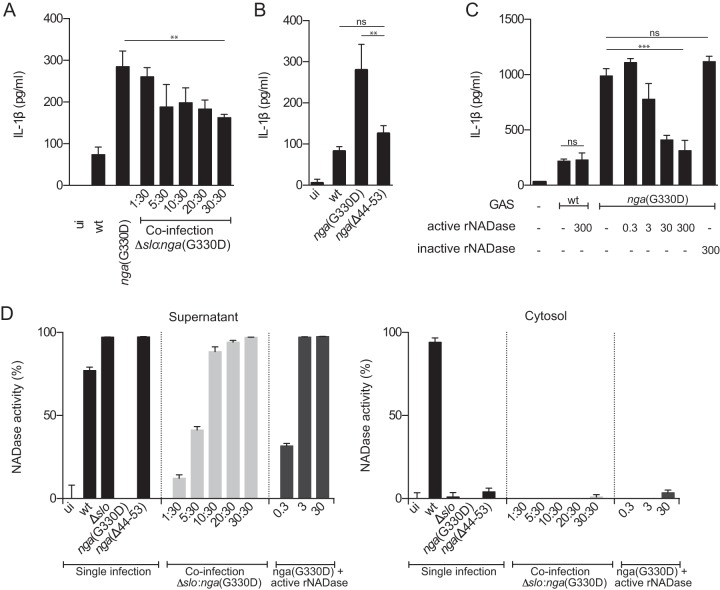
Extracellularly located, nontranslocated NADase regulates IL-1β levels. LPS-primed BMDMs were treated as follows: coinfected with Δ*slo* (MOI of 1 to 30) and *nga*(G330D) GAS (MOI of 30) (A), infected with wt, *nga*(G330D), or *nga*(Δ44-53) GAS (B), infected with wt or *nga*(G330D) GAS in the presence of active or inactive (G330D) recombinant NADase (rNADase) (0.3 to 300 nM) (C). Levels of IL-1β were analyzed by ELISA. (D) NADase activity was assessed by measuring NAD^+^ consumption in the supernatant and cytosolic fractions of infected (as above) and fractionated BMDMs. Active rNADase was titrated into *nga*(G330D) bacteria; infections at 0.3 to 30 nM. Graphs show means plus SD for triplicate samples and are representative of at least three independent experiments. Values that are significantly different are indicated by asterisks as follows: **, *P* ≤ 0.01; ***, *P* ≤ 0.001.

GAS adhering to host cells can perform CMT, i.e., allow SLO-dependent translocation of NADase across the cell membrane. However, because nonadherent bacteria, and to some extent also adherent bacteria (15), secrete these toxins into the extracellular milieu, it is reasonable to assume that under any infectious condition, NADase is present both intra- and extracellularly. Thus, we next set out to investigate where NADase toxin is localized when acting as an immune modulator. We took advantage of the fact that CMT can occur only when SLO and NADase are expressed from the same bacterium (15) and set up coinfection experiments using a Δ*slo* mutant strain (deficient for SLO expression, secreting active NADase) and a *nga*(G330D) mutant strain (proficient for SLO expression, secreting enzymatically dead NADase). This combined infection allows SLO-dependent inflammasome activation but does not support CMT, and accordingly, active NADase secreted by the Δ*slo* strain is not translocated but remains extracellular. Remarkably, introducing increasing amounts of active and extracellular NADase (through increasing numbers of Δ*slo* bacteria) into *nga*(G330D) bacterial infections caused a decrease in IL-1β levels (Fig. 3A), while cytotoxicity levels were only slightly but not significantly affected (Fig. S6A). These findings suggest that extracellularly located NADase is responsible for the negative regulation of IL-1β secretion during wt GAS infections. To further corroborate this unexpected result, we infected BMDMs with the *nga*(Δ44-53) mutant strain which expresses SLO like wt bacteria (Fig. S1) but harbors a 10-residue deletion from positions 44 to 53 in the NADase translocation domain, rendering the toxin deficient for CMT while unaffected in NADase expression levels (Fig. S1) or enzymatic activity (21). The amount of IL-1β released from BMDMs infected with this strain was similar to that released from wt-infected cells (Fig. 3B), further indicating that NADase exerts its function from the extracellular compartment. A third line of evidence supporting the notion that nontranslocated NADase modulates inflammasome-dependent IL-1β levels was generated by adding increasing amounts of pure active recombinant NADase (rNADase) into BMDM infections with *nga*(G330D) bacteria, resulting in a dose-dependent decrease of the IL-1β levels (Fig. 3C). A similar addition of inactive rNADase (harboring the G330D mutation) did not affect the levels of secreted IL-1β (Fig. 3C). Notably, and in agreement with our conclusion that streptococcal NADase is not regulating IL-1α release from infected cells (Fig. 1D), the levels of released IL-1α were also unaffected by the addition of either recombinant toxin (Fig. S6B), suggesting that NADase does not have a general effect on cytokine production or release during GAS infection.

10.1128/mBio.00756-17.6 FIG S6  Effects of extracellular NADase on LDH and cytokine release from infected BMDMs. (A) LPS**-**primed BMDMs were infected with wt or *nga*(G330D) GAS (MOI of 30) or coinfected with Δ*slo* (MOI of 1 to 30) and *nga*(G330D) (MOI of 30) bacteria. LDH release into the supernatant was measured to determine cytotoxicity. Values were normalized to the values for LDH release induced by wt GAS infection. The graph shows means ± SD for triplicate samples and is representative of three independent experiments. (B) BMDMs were primed with LPS followed by infection with wt or *nga*(G330D) bacteria in the presence or absence of rNADase (0.3 to 300 nM). IL-1α in the supernatant was determined by CBA. The graph shows means ± SD for triplicate samples from one experiment. (C and D) IL-1β levels were measured upon titration of recombinant NADase (0.3 to 300 nM) into ATP (5 mM) and nigericin (10 µM) stimulation for 30 min of LPS-primed BMDMs. Graphs show means ± SD for triplicate samples and are representative of at least three independent experiments. Download FIG S6, EPS file, 2.1 MB. Copyright © 2017 Hancz et al. 2017 Hancz et al. This content is distributed under the terms of the Creative Commons Attribution 4.0 International license.

We were interested in investigating whether streptococcal NADase could be used to inhibit IL-1β release upon inflammasome activation induced by other stimuli than GAS infection. Thus, we titrated active rNADase into LPS-primed BMDMs in which the Nlrp3 inflammasome had been activated either by ATP (Fig. S6C) or nigericin (Fig. S6D) and measured the levels of released IL-1β. Surprisingly, cytokine release induced by these compounds was unaffected by the addition of rNADase, implying that NADase modulation of inflammasome output may be specific to GAS infection.

Although CMT occurs only when SLO and NADase are expressed from the same bacterium (15), it remained theoretically possible that extensive membrane damage in our system could allow for passive entry of NADase into the macrophage cytosol where it could exert its immune modulatory function. We therefore analyzed NADase activity, as assessed by NAD^+^ consumption, in the culture supernatant or cytosol of BMDMs after coinfection, infection with the *nga*(Δ44-53) mutant strain or infection with the *nga*(G330D) mutant supplemented with active rNADase (Fig. 3D). As expected, infection with wt GAS resulted in NADase activity in the macrophage cytosol as well as in the culture supernatant, demonstrating that NADase is translocated across the host cell membrane but is also released into the culture medium. In contrast, no cytosolic NADase activity could be detected after Δ*slo*::*nga*(G330D) coinfection, infection with *nga*(Δ44-53) or *nga*(G330D) infection supplemented with active rNADase, while all of these infections generated activity in the supernatant. Collectively, these data indicate that the observed immune regulatory effect of NADase can be ascribed to extracellular rather than translocated NADase, identifying a novel compartment from which this toxin may exert function.

### NADase regulates IL-1β levels without affecting caspase-1 activity or the levels of involved inflammasome components.

**FIG 4:**
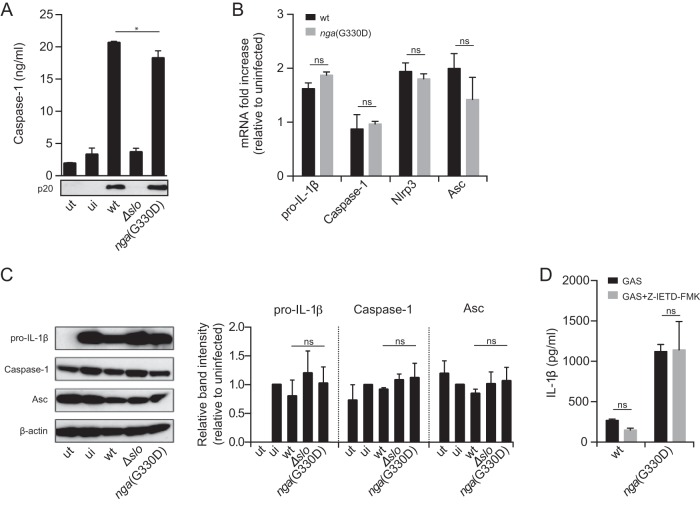
Streptococcal NADase regulates IL-1β levels without affecting caspase-1 activity or levels of involved inflammasome components. (A) LPS-primed BMDMs were infected with wt, Δ*slo*, or *nga*(G330D) GAS. and the level of active caspase-1 released into the supernatant was analyzed by ELISA or Western blotting. Values that are significantly different (*P* ≤ 0.05) are indicated by a bar and asterisk. ut, untreated cells; ui, LPS-primed uninfected cells. (B) mRNA levels of indicated inflammasome components were assessed by quantitative reverse transcription-PCR (RT-qPCR) following infection. Values are normalized to the values for LPS-primed, uninfected BMDMs. (C) Protein levels of pro-IL-1β, caspase-1, and ASC were investigated in LPS-primed and infected Nlrp3^−/−^ BMDMs to avoid IL-1β and caspase-1 processing and release and analyzed by Western blotting. Band intensity, relative to that of β-actin, was quantified and normalized to primed, uninfected cells. (D) IL-1β release upon infection in the presence of 20 μM Z-IETD-FMK (caspase-8 inhibitor) was measured by ELISA. The graphs in panels A, B, and D show means plus SD for triplicate samples and are representative of at least three independent experiments. The blot in panel A shows one representative blot of five independent experiments. Panel C shows one representative blot and means plus SD from three independent experiments.

Although the precise mechanistic details of how assembly of the Nlrp3 inflammasome is induced and leads to IL-1β release are still unclear, it is widely accepted that Nlrp3 inflammasome activation can be measured by detection of processed caspase-1. Interestingly, we were not able to detect differential caspase-1 activation in BMDMs infected with wt or *nga*(G330D) bacteria as measured by secreted active caspase-1 enzyme-linked immunosorbent assay (ELISA) or Western blotting (Fig. 4A) or a colorimetric assay using a substrate for active caspase-1 (data not shown), suggesting that the increased IL-1β levels observed in *nga*(G330D) bacterial infections are not caused by increased inflammasome activation *per se* by this strain. Moreover, we detected no alterations in the amounts of generated mRNA of pro-IL-1β, caspase-1, Nlrp3, or ASC in infected B6 BMDMs (Fig. 4B), indicating that transcriptional regulation of inflammasome components does not differ between cells infected by wt or *nga*(G330D) bacteria. In addition, we could not observe any differences in protein levels of pro-IL-1β, caspase-1, or ASC between BMDMs infected by wt or *nga*(G330D) bacteria (Fig. 4C); because analysis of intracellular levels of inflammasome proteins in cells undergoing inflammasome activation is complicated by the fact that activation mediates cleavage and secretion of these very components, this analysis was performed in Nlrp3-deficient BMDMs, which do not activate the inflammasome in response to GAS infection. Analysis in ASC-deficient BMDMs generated similar results (data not shown). These observations are in line with our data on IL-1α secretion (Fig. 1D), which similarly lead us to hypothesize that the detected difference in induced IL-1β secretion by our bacterial strains is likely due to regulation at a posttranscriptional level. Thus, we conclude that differential IL-1β levels are not due to variable transcriptional or translational regulation of inflammasome components.

Recent data demonstrate that activation of Nlrp3 inflammasomes may require caspase-8 in addition to caspase-1 and that caspase-8 can directly cleave IL-1β (40, 41). To investigate whether the increased levels of IL-1β induced by the *nga*(G330D) strain can be ascribed to differential involvement of caspase-8, we performed GAS infections in B6 BMDMs in the presence of an inhibitor of caspase-8 activity. As IL-1β secretion was unaffected by caspase-8 inhibition in both wt and *nga*(G330D) bacterial infection (Fig. 4D), our data suggest that neither inflammasome activation nor the regulation of IL-1β levels induced by GAS involve caspase-8 activity. Taken together, these data indicate that inhibition of inflammasome-dependent IL-1β release by NADase cannot be ascribed to differential activation of the inflammasome or to transcriptional or translational differences in inflammasome components.

## DISCUSSION

During recent decades, life-threatening invasive GAS disease has increased globally, due in particular to emergence of a highly virulent M1T1 clone. Remarkably, invasive M1T1 strains isolated after 1988 seem to almost exclusively be clonal derivatives of the same parental strain (6), and genetic analysis of isolates belonging to this clonal type have revealed the acquisition of a genomic region conferring increased expression of SLO and NADase (5). In addition, the M89 clade 3 strains which are currently emerging as a major cause of invasive disease carry the same *nga* promoter region as the globally spread M1T1 clone and similarly overexpress SLO and NADase (8, 31), suggesting that this promoter sequence constitutes a genetic signature for increased bacterial virulence. It has also been shown that, regardless of M type, strains expressing active NADase are overrepresented in invasive disease (42), likewise implying an important role for this toxin in GAS pathogenesis. An NADase-deficient mutant of an invasive M3 isolate was shown to have reduced virulence compared to the parent strain in a mouse model of sepsis after intraperitoneal challenge and in a soft tissue infection model, adding further support to a role for NADase in pathogenesis (18). In the present study, we used a GAS strain representative of the M1T1 clonal type and identified a novel role for active NADase as a negative regulator of SLO-mediated inflammasome-dependent IL-1β release, further contributing to our understanding of the increased virulence exhibited by these strains. Unexpectedly, we were able to assign this role in immune evasion to extracellular NADase, ascribing a function to the nontranslocated fraction of this toxin. Thus, herein we describe two novel findings pertaining to streptococcal NADase. First, this toxin may play a role in immune evasion by inhibiting inflammasome output from immune cells. Second, this effect is exerted by toxin present in the extracellular compartment.

**FIG 5:**
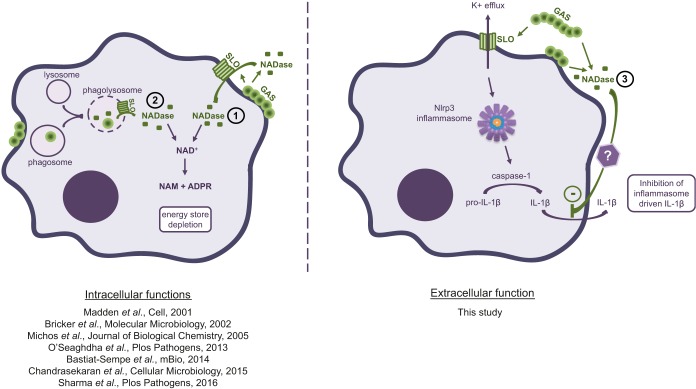
Streptococcal NADase has different functions depending on toxin localization. In function 1 (shown by an circled 1), extracellularly located, adherent bacteria translocate NADase into the host cell cytosol via CMT. In function 2, phagocytosed GAS residing in the phagolysosome also translocates NADase to the cytosol similarly to adherent bacteria. NADase localized intracellularly hydrolyze NAD^+^, leading to energy store depletion of host cells. In function 3, as shown in this study, NADase secreted to the extracellular milieu has the ability to inhibit the secretion of Nlrp3 inflammasome-driven IL-1β, identifying a novel function as well as a previously unrecognized functional niche for NADase toxin.

Previous studies have shown that bacteria adhering to the surfaces of host cells, as well as bacteria that have been phagocytosed, may translocate NADase across the plasma or phagosomal membrane into the host cell cytosol (Fig. 5). In infected tissues, however, where it can be expected that bacteria will be both adherent and nonadherent to host cells, NADase will be translocated to the interior of these cells as well as released into the extracellular compartment. The role of intracellular NADase has been extensively investigated, and its effects include inhibition of GAS internalization, cell death due to depletion of intracellular NAD^+^ and ATP, and prevention of autophagosomal maturation, acidification of phagolysosomes, and trafficking of intracellular GAS to lysosomes (17, 19–22, 24). Our results do not contradict these previous findings but add a novel aspect to our understanding of the position of the NADase toxin in GAS pathogenesis and imply that its functions should be considered not only in situations when bacterial adhesion is expected to occur or after translocation into host cells (Fig. 5).

The role of IL-1β in human GAS infections has not been extensively investigated. However, as patients treated with the IL-1R antagonist anakinra for different inflammatory syndromes run a significantly increased risk of developing streptococcal necrotizing fasciitis, it seems clear that IL-1 signaling is essential in the control of these invasive and often lethal GAS infections (26). Murine models of GAS infection paint a complex picture of the function for IL-1β (43, 44). Indeed, while IL-1R^−/−^ mice are highly susceptible to GAS infection and succumb to bacteremia (43), implying that IL-1β (and/or IL-1α) is important to inhibit bacterial outgrowth, it has also been shown that in a panel of mouse strains, increased susceptibility to GAS infection links genetically to a locus on chromosome 2 (Chr2) harboring genes of the IL-1 network and correlates with increased levels of IL-1β and bacterial expansion in infected animals (44). Taken together, these studies suggest that dysregulated IL-1β responses are detrimental to infection control, including both overly vigorous and restrictive responses, and that appropriate levels induced during infection are crucial for the survival of an infected host. It has also been shown that mice deficient in type I interferon signaling cannot limit IL-1β levels during GAS infection and succumb to cytokine-driven pathology in the absence of bacterial outgrowth in the infected animal, underlining the powerful inflammatory effects of this cytokine as well as its functional complexity in infection control (43). These studies concurrently highlight the importance of the ability for successful pathogens to modulate induced immune responses, as exemplified by the findings described in this work.

Many bacterial pathogens secreting pore-forming toxins, including GAS, *Streptococcus pneumoniae*, *Staphylococcus aureus*, and *Listeria monocytogenes*, activate the Nlrp3 inflammasome through K^+^ efflux across the plasma membrane induced by these cytolysins (45–47). Our findings indicate that GAS has evolved strategies to directly inhibit the levels of cytolysin-induced inflammasome-dependent IL-1β, although the exact regulating mechanism remains to be defined. It is also unclear whether other cytolysin-expressing pathogens employ mechanisms to inhibit IL-1β levels induced by these toxins. Interestingly, and in contrast to previously described microbial mechanisms of regulating or evading inflammasome activation (48), NADase-dependent inhibition of IL-1β by GAS cannot be explained by actual modulation of inflammasome activation or accessibility of inflammasome components (Fig. 4). IL-1β is a leaderless protein, and several mechanisms have been suggested to govern its release from cells. These mechanisms include contained release in multivesicular bodies or exosomes, secretory lysosomes or autophagy, by active transporters across the plasma membrane or passive release from dying cells (49), and most recently through membrane gasdermin D pores (50). It remains possible that GAS NADase regulates secretion *per se* of mature IL-1β, interfering with the secretion pathway at a step subsequent to processing of pro-IL-1β. Of note, secretion of mature IL-1β induced by wt as well as NADase-deficient bacteria is independent of remodeling of the actin cytoskeleton, as inhibition of actin remodeling had no effect on the IL-1β levels provoked by either strain (Fig. 2), suggesting that the release pathway(s) employed does not require actin filaments for transport. In addition, and in contrast to several other Nlrp3-activating stimuli (39), these data also suggest that actin remodeling is not required for SLO-mediated Nlrp3 inflammasome activation. Our data further indicate that IL-1β is actively transported from GAS-infected macrophages and not merely released as a consequence of cell death, as in the absence of transcriptional and protein level alterations of relevant inflammasome components, no differences in cell viability were observed after infection with the wt or NADase-deficient strains, while IL-1β levels were variable (Fig. 1). Interestingly, a recent study suggests that the streptococcal protease SpeB may serve to generate inflammasome-independent IL-1β during GAS infection under caspase-1-deficient conditions *in vitro* and *in vivo* and that this proteolysis might drive the hypervirulence conversion observed during infection with certain GAS strains (26). We observed no SpeB-dependent IL-1β secretion *in vitro* regardless of the presence or absence of caspase-1. The reasons for this discrepancy are not known but may pertain to strain-specific differences as well as differences in experimental conditions.

In summary, our results demonstrate that streptococcal NADase inhibits the macrophage production of inflammasome-dependent IL-1β. Surprisingly, this effect of NADase does not require translocation into the host cell cytosol but is apparently exerted extracellularly, identifying a new functional niche for this toxin. In particular, our data indicate that NADase has an important function in immune evasion by GAS, making it of interest to further explore the mechanistic basis for NADase modulation of IL-1β as well as investigate whether this property of NADase may, at least partially, explain why enhanced expression of this toxin is correlated with bacterial virulence.

## MATERIALS AND METHODS

### Bacterial strains and growth conditions.

The wild-type (wt) GAS strain 854 used in this study is an M type 1 (M1) strain isolated from a patient with a retroperitoneal abscess (51). The isogenic Δ*slo*, *nga*(G330D), and *nga*(Δ44-53) mutant strains have been described previously (21). A mutant deficient in production of SpeB (Δ*speB*) was constructed in strain 854 by insertion of the Ωkm-2 interposon into the chromosomal *speB* locus as described previously (52). Bacteria were grown in Todd-Hewitt broth supplemented with 0.5% yeast extract (THY) in 5% CO_2_ at 37°C. Overnight cultures were reinoculated in fresh THY and grown to late exponential phase (optical density at 600 nm [OD_600_] of 1.1 to 1.3), collected by centrifugation, washed with phosphate-buffered saline (PBS), and diluted before use.

### Mice.

All genetically modified mouse strains (caspase-1/-11^−/−^ [53], caspase-11^−/−^ [54], Nlrp3^−/−^ [55], Asc^−/−^ [56], Nlrc4^−/−^ [56], Naip5^−/−^ [57]) had a C57BL/6 (B6) background and were bred and maintained at the animal facility of the Biomedical Center, Lund University. B6 mice were bred in-house. Genetically modified mouse strains were kindly provided by Russell E. Vance, Bengt Johansson-Lindbom, and Genentech Inc. All animal experiments were conducted in accordance with protocols approved by the Lund/Malmö Animal Ethics Committee.

### Generation of bone marrow-derived macrophages.

Bone marrow was isolated from murine femurs, and progenitor cells were differentiated into bone marrow-derived macrophages (BMDMs) for 7 days in RPMI 1640 (Gibco) supplemented with 10% fetal bovine serum (FBS) (Sigma), 2.5 mM l-glutamine, and macrophage colony-stimulating factor at 37°C in 5% CO_2_.

### *In vitro* bacterial infections of BMDMs.

BMDMs were seeded at 7.5 × 10^4^ cells per well in 96-well plates (unless otherwise stated), primed with 1 µg/ml lipopolysaccharide (LPS) (Sigma) for 15 h, and infected at a multiplicity of infection (MOI) of 30, centrifuged for 5 min (300 × *g*), and incubated for 1.5 h. At this time point, the bacterial suspension was replaced with fresh medium containing 300 µg/ml gentamicin (Sigma) to kill extracellular bacteria, and the BMDMs were incubated for another 3 h. When indicated, cells were infected in the presence of 20 µM caspase-8 inhibitor: Z-IETD-FMK (BD Biosciences) or 0.3 to 300 nM active or inactive recombinant NADase (24).

### Differentiation and infection of THP-1 cells.

THP-1 cells were cultured in RPMI 1640 (Gibco) supplemented with 10% FBS (Sigma), 2 mM l-glutamine, 1 mM sodium pyruvate, 5 mM HEPES, 4.5 mg/ml d-glucose, and 0.05 mM 2-mercaptoethanol at 37°C in 5% CO_2_. THP-1 cells were differentiated into macrophages by stimulation with 5 ng/ml phorbol 12-myristate 13-acetate for 72 h, adding 5 ng/ml LPS for the last 24 h. GAS infection of THP-1 cells was performed as described for BMDMs above.

### Cytokine and caspase-1 analysis.

Supernatants from infected BMDMs or THP-1 cells were cleared from debris by centrifugation (300 × *g* for 5 min) and analyzed using IL-1β (BD Biosciences) or caspase-1 (AdipoGen) ELISA kits or IL-1α cytometric bead array (BD Biosciences) according to the manufacturer’s instructions. Notably, we have detected significant fluctuations in absolute IL-1β levels in our experiments, likely due to features of the ELISA kit. Importantly, the ratio of IL-1β induced by different treatments remains stable throughout all experiments performed.

### Cytotoxicity assays.

Macrophage cytotoxicity was assessed by measuring lactate dehydrogenase (LDH) release from infected BMDMs. Supernatants were cleared by centrifugation as described above and analyzed using CytoTox 96 assay (Promega) according to the manufacturer’s instructions. Uninfected cells were used to determine the background level of LDH release, and lysed, uninfected cells were used as a reference for maximal cytotoxicity.

### Reverse transcribed quantitative PCR.

For gene expression analysis, infections were performed as described above using 5 × 10^5^ BMDMs in 24-well plates. After 1.5 h, the bacterial suspension was removed, and total RNA was isolated from the BMDMs using the SV total RNA isolation system (Promega). The GoScript reverse transcription system (Promega) was used to generate cDNA, and specific gene transcripts were quantified using Sso Fast EvaGreen supermix (Bio-Rad) with the following primer pairs. For pro-IL-1β, the forward (Fwd) primer was GGTCAAAGGTTTGGAAGCAG, and the reverse (Rev) primer was TGTGAAATGCCACCTTTTGA. For caspase-1, the Fwd primer was TGGAAATGTGCCATCTTCTTT, and the Rev primer was TCAGCTCCATCAGCTGAAAC. For Nlrp3, the Fwd primer was AAGTAAGGCCGGAATTCACC, and the Rev primer was AAATGCCTTGGGAGACTCA. For Asc, the Fwd primer was GCTGGTCCACAAAGTGTCCT, and the Rev primer was GAGCAGCTGCAAACGACTAA. For glyceraldehyde-3-phosphate dehydrogenase (GAPDH), the Fwd primer was TTGATGGCAACAATCT, and the Rev primer was CGTCCCGTAGACAAAA. The relative change in transcript levels upon infection was calculated using the ΔΔ*C*_*T*_ method with values normalized to GAPDH.

### Protein separation and immunoblot assays.

For Western blot assays, bacterial infections were performed using 5 × 10^5^ BMDMs from strains with the indicated genotype in 24-well plates. Cell lysates were prepared by the addition of Nonidet P-40 lysis buffer (150 mM sodium chloride, 1% NP-40, 50 mM Tris, 10× complete protease inhibitor cocktail [Roche]) to total cells after 1.5 h of infection. To detect cleaved caspase-1, following 1.5-h infection and 3-h incubation in fresh serum-free medium, supernatants were precipitated with 10% trichloroacetic acid (Sigma) in the presence of 0.01% bovine serum albumin (BSA) (Sigma) for 30 min. Pellets collected by centrifugation (16,000 × *g*, 15 min) were washed with acetone, dried, and resuspended in lithium dodecyl sulfate (LDS) sample buffer (Life Technologies) containing sample reducing agent (Life Technologies). To compare the levels of secreted NADase and SLO by isogenic strains, overnight cultures were reinoculated in fresh THY and grown to late exponential phase (OD_600_ of 1.15). Supernatant was cleared by centrifugation and mixed with sample buffer and sample reducing agent. Bacterial cell lysate was prepared by the addition of sample buffer and reducing agent to pelleted bacteria followed by boiling (95°C) for 5 min. Samples were separated by SDS-PAGE using NuPAGE 12% bis-Tris gels (Life Technologies) under reducing conditions and transferred to Hybond polyvinylidene difluoride (PVDF) membranes (GE Healthcare). For detection, the following primary and horseradish peroxidase (HRP)-conjugated secondary antibodies were used: anti-IL-1β (catalog no. AF-401; R&D Systems), anti-caspase-1 p20 (catalog no. AG-20B-0042; AdipoGen), anti-ASC (catalog no. sc-22514-R; Santa Cruz Biotechnology), anti-β-actin (catalog no. PA1-21167; Thermo Scientific), anti-streptolysin O (catalog no. ab188539; Abcam), anti-NADase (catalog no. 64-005; BioAcademia), anti-M1 protein (58), rabbit anti-goat IgG (catalog no. 61-1620; Thermo Scientific), donkey anti-mouse IgG (catalog no. 715-036-151; Jackson Immuno Research), and goat anti-rabbit IgG (catalog no. 111-035-144; Jackson Immuno Research). Stained membranes were incubated in Clarity Western ECL blotting substrate for chemiluminescence (Bio-Rad) and developed. Bands were quantified with ChemiDoc imaging system (Bio-Rad) using Quantity One and Image Lab software.

### Inhibition of phagocytosis.

To inhibit phagocytosis, primed BMDMs were incubated with 5 µg/ml cytochalasin D (Sigma) (CytD) for 45 min prior to infection. The infection was performed as described above in the presence of CytD. The proportion of internalized bacteria was determined following lysis of infected BMDMs using 0.1% Triton X (Sigma). Lysed samples were plated on blood agar plates to determine the number of intracellular bacteria that was correlated with the total number of bacteria used for infection. Proper CytD activity was confirmed using CytoSelect 96-well phagocytosis assay (zymosan substrate) (Cell Biolabs) according to the manufacturer’s instructions.

### Measurement of NADase activity.

For NADase activity measurements, 3 × 10^5^ BMDMs were infected in 24-well plates as described above. For the last 20 min of the incubation with GAS, 500 µg/ml gentamicin (Sigma) was added to kill bacteria and thus prevent NADase production during sample preparation. Cell fractionation and NADase activity measurements were performed as described previously (17). Briefly, supernatants from infected BMDMs were collected and cleared of bacteria and cell debris by centrifugation. BMDMs were scraped with ice-cold PBS, pooled with cell debris, and lysed in sterile water. The whole-cell lysate was cleared from the membrane fraction by centrifugation at 20,300 × *g* for 5 min to yield the cytosolic fraction. To measure NADase activity, NAD^+^ (Sigma) was added at a final concentration of 0.67 mM to the culture supernatant or cytosolic fraction, and the reaction mixtures were incubated at 37°C for 3 h. To develop reactions, NaOH (2 N) was added, and the plates were incubated in the dark at room temperature for 1 h. Samples were read on a Varioskan LUX multimode reader (Thermo Scientific) at 360-nm excitation/530-nm emission. The calculated fluorescence value reflects the inverse relationship between the fluorescence of the remaining NAD^+^ and NADase activity. Complete hydrolysis of added NAD^+^ was set to correspond to 100% NADase activity.

### Data processing and statistical analysis.

Statistical calculations were performed using one or two-way analysis of variance (ANOVA). *P* values are indicated by asterisks as follows: *, *P* ≤ 0.05; **, *P* ≤ 0.01; ***, *P* ≤ 0.001.
